# 2020 ESC Guidelines on Sports Cardiology: Impact of CMR Criteria on Return-to-Play Clearance After Acute Myocarditis

**DOI:** 10.3390/jcdd12120469

**Published:** 2025-11-29

**Authors:** Carlo Maria Gallinoro, Alessandra Scatteia, Dario Catapano, Carmine Emanuele Pascale, Giuseppe Russo, Franca Di Meglio, Santo Dellegrottaglie

**Affiliations:** 1 Advanced Cardiovascular Imaging and Sports Cardiology Unit, Clinica Villa dei Fiori, 80011 Acerra, Italy; 2 Cardiology and Cardiovascular Intensive Care Unit, P.O. S. Giuseppe Moscati, 81031 Aversa, Italy; 3 Radiology Unit, Clinica Villa dei Fiori, 80011 Acerra, Italy; 4 Public Health Department, University of Naples Federico II, 80131 Naples, Italy

**Keywords:** acute myocarditis, sport cardiology, return-to-play, CMR, ESC guidelines on Sports Cardiology, myocarditis, athletes

## Abstract

Cardiovascular magnetic resonance (CMR) imaging is a key component of current diagnostic pathways in subjects with acute myocarditis. The 2020 ESC Guidelines on Sports Cardiology recommend athletes with acute myocarditis to abstain from sports during the recovery phase from inflammation and to undergo comprehensive evaluation—including CMR—before safely returning to play. This retrospective study analyzed 95 non-competitive athletes presenting with acute myocarditis and evaluated by initial and repeated CMRs. CMR exams assessed myocardial inflammation, edema, and scarring as defined based on the updated Lake Louise criteria. As per 2020 ESC Guidelines, eligibility was granted by excluding extensive myocardial damage. Initial CMR showed 84% positive STIR (edema) and 79% with LGE ≥ 3 segments. After 3–6 months, STIR positivity dropped to 12%, LGE extent remained globally stable, but with some reduction in 42%. Few experienced recurrent myocarditis or LVEF decline; 24% met return-to-play criteria by repeated CMR. Our study shows that few non-competitive athletes recovering from acute myocarditis meet ESC CMR criteria to resume competitive sports at prescribed follow-up evaluation. The long-term prognostic value of CMR markers like LGE and edema remains unclear, highlighting the need for further research to refine return-to-play guidelines and ensure athlete safety.

## 1. Introduction

Myocarditis is an inflammatory disease of the heart muscle (myocardium) that can stem from a variety of causes, with a wide spectrum of potential clinical presentations [1]. The vast majority of cases are presumably post-viral or are labeled as idiopathic and lead to complete recovery. Of note, acute myocarditis should be included in the differential diagnosis with some cardiomyopathies, such as desmoplakinopathies, possibly presenting with acute hot phases characterized by chest pain, myocardial inflammation, and cell death accompanied by the release of troponins [2].

Cardiovascular magnetic resonance (CMR) imaging is crucial for diagnosing myocarditis, providing detailed functional assessment and myocardial tissue characterization, including effective detection of inflammation, edema, fibrosis, and necrosis. The recent 2025 ESC (European Society of Cardiology) Guidelines for the management of myocarditis and pericarditis have established CMR as equivalent to EMB for the proven diagnosis of acute myocarditis in low-to-intermediate risk cases [3]. By referring to the updated Lake Louise criteria, CMR findings enable precise assessment of myocarditis presence and severity, guiding treatment, and monitoring the course of the disease at myocardial tissue level [3,4].

Myocarditis has recently become a significant focus within the realm of Sports Cardiology due to its potential impact on athletes’ health and the heightened risk of severe cardiac events during strenuous exercise [5]. Given the potentially severe consequences of this inflammatory condition, national and international scientific societies have developed rigorous return-to-play protocols to ensure athletes’ safety [6,7]. The proposed criteria emphasize a comprehensive assessment to confirm absence of symptoms, resolution of inflammation, and stable cardiac function before an athlete resumes competitive activity [6,7]. The 2020 ESC Guidelines on Sports Cardiology and Exercise in Patients with Cardiovascular Disease established formal criteria for return to sports for both competitive and non-competitive athletes who have experienced acute myocarditis [6]. Again, CMR deserves a relevant role, given its capability to provide detailed images of myocardial damage, revealing any initial or lingering area of edema, fibrosis, or scar. The rationale behind the recommended comprehensive evaluation was to guide decisions regarding athletes’ participation in competitive sports giving priority to short-term and long-term health by minimizing the risk of life-threatening events.

This study aimed to collect data on subjects involved in non-competitive sport activity deemed suitable to return-to-play after experiencing acute myocarditis, with a particular focus on CMR criteria as a key determinant of eligibility.

## 2. Materials and Methods

### 2.1. Study Subjects

Data on non-competitive athletes involved in any sport activity referred to our CMR laboratory over a period of 3.5 years (between January 2021 and October 2024) for clinical suspicion of acute myocarditis were retrospectively collected. In this study, we defined non-competitive athletes as subjects who engage in structured physical exercise or sports for at least 4 hours per week and who do not participate in competitions. Clinical data such as age, traditional cardiovascular risk factors, family history, and levels of troponin were recorded. Only patients with at least a second CMR exam performed at follow-up were included, while patients with low-quality CMR images or incomplete CMR protocol were excluded. Written informed consent was obtained from all patients before undergoing any CMR scan

### 2.2. Cardiac Magnetic Resonance

CMR exams were performed in clinically stable patients using a 1.5 Tesla clinical scanner (Philips Achieva; Phillips Healthcare, the Netherlands) equipped with a dedicated cardiac software and a multi-channel phased-array coil system.

For each study, the CMR protocol included the acquisition of multiple series of ECG-gated images, obtained during short breath-hold periods in standard cardiac long-axis (4-chamber, 2-chamber, and 3-chamber) views and short-axis views, to completely cover the left ventricle (LV) from the atrio-ventricular plane to the apex. The following scan imaging sequences were included: balanced steady-state free-procession (b-SSFP) cine images, obtained for cardiac function evaluation; T2-weighted short tau inversion recovery (STIR) images, focusing on detection of myocardial edema; late gadolinium enhancement (LGE) post-contrast T1-weighted inversion recovery images, obtained 10–20 min after i.v. administration of 0.1 mml/kg of gadobutrol (Gadovist, Bayer Healthcare, Berlin, Germany) to asses LV areas of signal hyperenhancement as expression of acute myocardial damage or chronic scar/fibrosis. As part of our standard protocol applied in cases with suspected myocarditis, additional cine b-SSFP-images were obtained 1–3 min after contrast injection, to detect areas of high signal intensity that represent LV myocardial hyperemia [8]. In some cases, T1 mapping and T2 mapping images were also acquired, using the MOLLI (Modified Look-Locker Inversion Recovery) and GraSE (Gradient and Spin Echo) sequences, respectively. Typical scanning parameters selected for image acquisition in our CMR Laboratory are reported elsewhere [9].

### 2.3. CMR Image Analysis

The CMR imaging analysis was conducted offline by an expert CMR reader (holding Level III EACVI certification) using commercially available software (CVi42, Circle Cardiovascular Imaging Inc., Calgary, AB, Canada). LV volumes and ejection fraction were measured by tracing endocardial contours on end-diastolic and end-systolic short-axis cine images, and volume values were indexed to body surface area [9,10]. The presence and extent of edema on T2-weighted images and hyperemia on post-contrast cine SSFP images were qualitatively assessed. Using a 16-segment LV model, occurrence of non-ischemic LGE was described and reported as the number of involved LV segments. The diagnosis of myocarditis was established according to the 2018 updated Lake Louise criteria, which serve as a standard for identifying inflammatory myocardial diseases based on specific CMR findings [4]. Acute myocarditis was suggested by the coexistence of areas showing high signal intensity on T2-weighted STIR images and corresponding areas of non-ischemic LGE, indicative of active myocardial inflammation and damage. In case of negative T2-weighted images, findings on T1 mapping and/or post-contrast cine images were used in order to confirm the diagnosis of myocarditis. On the other hand, the identification of non-ischemic LGE without accompanying myocardial edema on T2-weighted images suggested either prior episodes of myocarditis or alternative forms of cardiomyopathy, with differentiation being guided by the patient’s clinical presentation and medical history. At follow-up CMR, a reduction, stability, or progression of LGE extent was assessed based on the number of myocardial segments affected.

### 2.4. Sports Eligibility Criteria

According to the 2020 ESC Guidelines on Sports Cardiology, specific CMR criteria must be satisfied for an athlete to be cleared for eligibility after acute myocarditis. These criteria include normal LV ejection fraction and the absence of myocardial edema, which indicates no signs of acute inflammation. Another crucial requirement is the absence of extensive LGE, defined as LGE > 20% of total LV mass. In our analysis, to maintain consistency and clinical applicability, LGE extent was defined by the number of LV segments involved, according to the cut-off recommended by the 2023 Italian COCIS Guidelines for return to competitive sports in athletes recovering from acute myocarditis [7]. Extensive LGE is associated with widespread myocardial damage, which can be indicative of more severe underlying heart conditions and worse prognosis [6].

### 2.5. Statistical Analysis

Continuous variables were expressed as mean, confidence intervals (ICs), standard deviations (SDs), minimum and maximum value, and variance when normally distributed. Categorical variables were expressed as percentages of the total population and compared using the χ^2^ test. The differences observed between the two scans were analyzed using a one-way Analysis of Variance (ANOVA) to determine whether there were statistically significant differences in the means of dependent variables (age, end-diastolic volume index, ejection fraction, LV segments involvement) among the group. A *p*-value < 0.05 was considered statistically significant. All statistical analyses were performed using the SPSS V. 30.0 statistical package

## 3. Results

**Figure 1:**
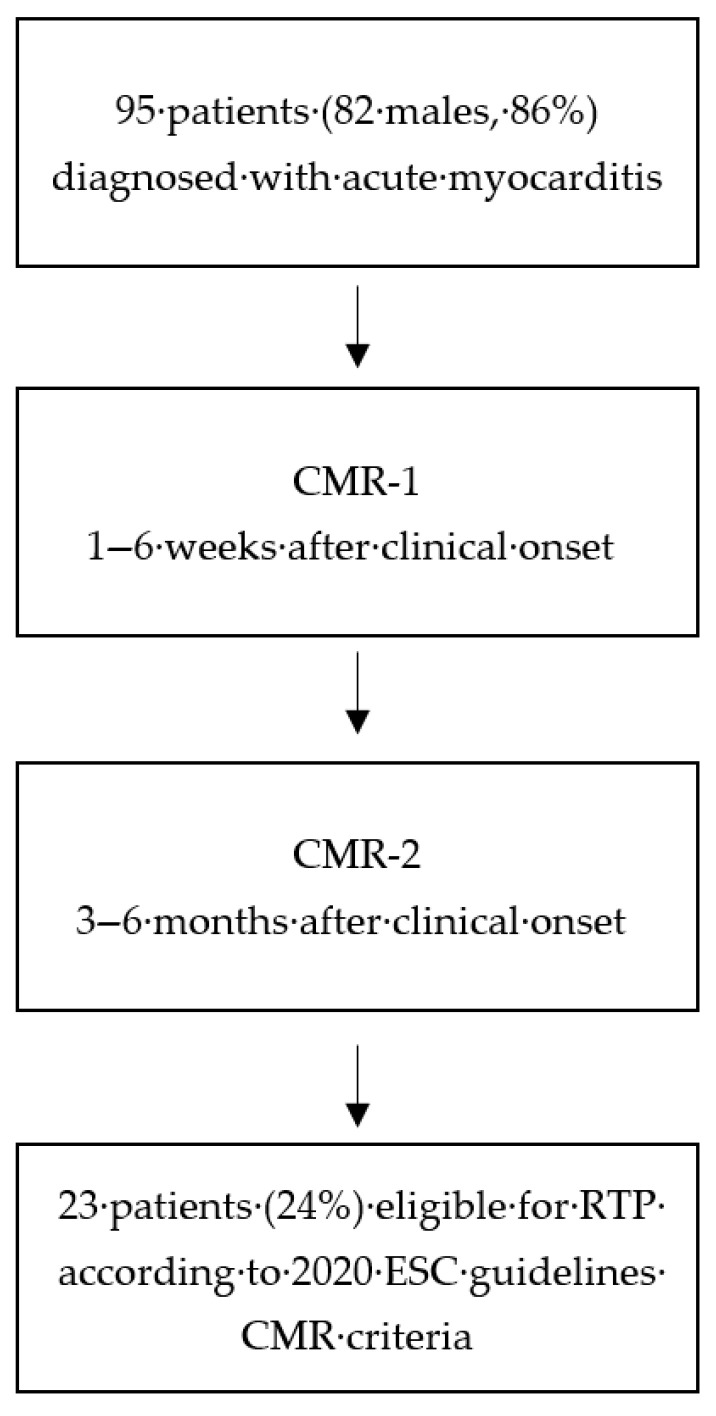
Overall study flow.

This study included a cohort of 95 patients (82 males, 86%) with an average age of 25 years ± 10 years (range 15–45 years) diagnosed with acute myocarditis with no complicated or high-risk clinical presentation. Diagnosis of suspected acute myocarditis was confirmed by an initial CMR scan (CMR-1) performed 1–6 weeks after clinical onset. Follow-up CMR scan (CMR-2) was performed after an additional period of 3–6 months of restricted physical activity, as indicated by the 2020 ESC Guidelines on Sports Cardiology. Overall study flow is reported in Figure 1. Variables considered as primary parameters included the time elapsed from clinical onset to the CMR evaluation, ejection fraction variation, T2-weighted STIR positivity/negativity, LGE extent and distribution (describing involvement of interventricular septum), and troponin levels (Table 1). During the observation period, two patients (2%) experienced recurrent acute myocarditis, seven patients (7%) were diagnosed with or suspected to have cardiomyopathy, one patient (1%) received an implantable cardioverter–defibrillator (ICD), and no patient experienced SCD or major arrhythmias.

### 3.1. Initial CMR Scan (CMR-1)

**Figure 2:**
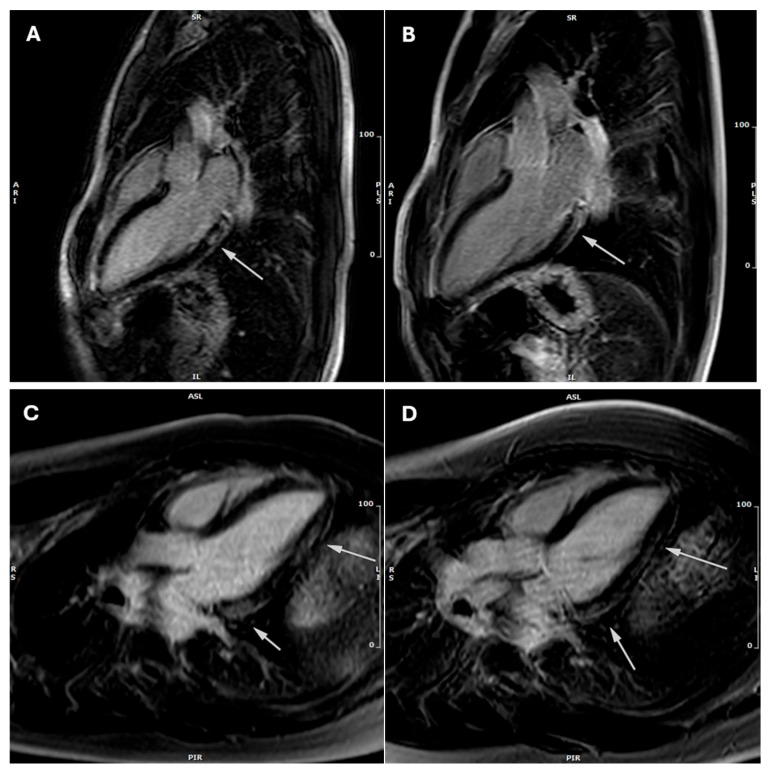
Three-chamber late gadolinium enhancement (LGE) cardiac magnetic resonance (CMR) images of two athletes at baseline and follow-up. (**A**) LGE-positive image from the first athlete at baseline CMR-1 showing limited involvement of the basal inferolateral wall of the left ventricle. (**B**) LGE-negative image from the same athlete at follow-up CMR-2 demonstrating resolution of LGE. (**C**) LGE-positive image from a second athlete at baseline CMR-1 displaying extensive involvement of the inferolateral wall of the left ventricle. (**D**) LGE-positive follow-up image of the second athlete at CMR-2 showing unchanged extent of LGE. The arrows delineate the regions exhibiting LGE.

At the first CMR, conducted on average one month after clinical onset, 84% (80 patients) presented with positive (+) STIR findings (Figure 2), while 16% (15 patients) were STIR negative (−). Two patients exhibited an LV ejection fraction below 55%. The mean value of the LV ejection fraction is 65% (95% CI: [64%, 67%]; SD: ±6%; min: 40%, max: 79%; variance: 153). Only a few patients [5] showed a mildly dilated left ventricle, with a mean EDVi of 78 mL/m^2^ (95% CI: [76, 81]; SD: ±12; min: 49, max: 109; variance: 67). The mean extent of the segments involved by LGE was 6 (95% CI: [5, 7]; SD: ±4; min: 1, max: 16; variance: 14). LGE ≥ 3 segments was noted in 79% of patients (75 individuals), with septal involvement observed in 10 cases.

### 3.2. Follow-Up CMR Scan (CMR-2)

Follow-up CMR was performed 3–6 months after clinical onset. Only 12 patients (13%; *p* ≤ 0.001 vs. CMR-1) showed positive STIR images. A total of 72% percent of patients (68 individuals) still exhibited LGE≥ 3 segments. LGE extent (mean: 5; 95% CI: [4, 6]; SD: ±4; min. 0, max. 16; variance: 13; *p* = 0.1 vs. CMR-1) and occurrence of LGE involving the septum (11%, 11 patients; *p* = 0.81 vs. CMR-1) remained substantially unchanged. No differences were observed in LV ejection fraction (mean: 66%; 95% CI: [64%, 67%]; SD: ±6%; min., max.; variance: 34%; *p* = 0.67) and end-diastolic volumes compared to CMR-1 (mean: 78 mL/m^2^; 95% CI: [76, 81]; SD: ±11; variance: 139; *p* = 0.9). Eventually, two patients experienced deterioration of LV ejection fraction to values below normality. None of the patients presenting with a septal LGE pattern were deemed eligible for return-to-play.

**Figure 3:**
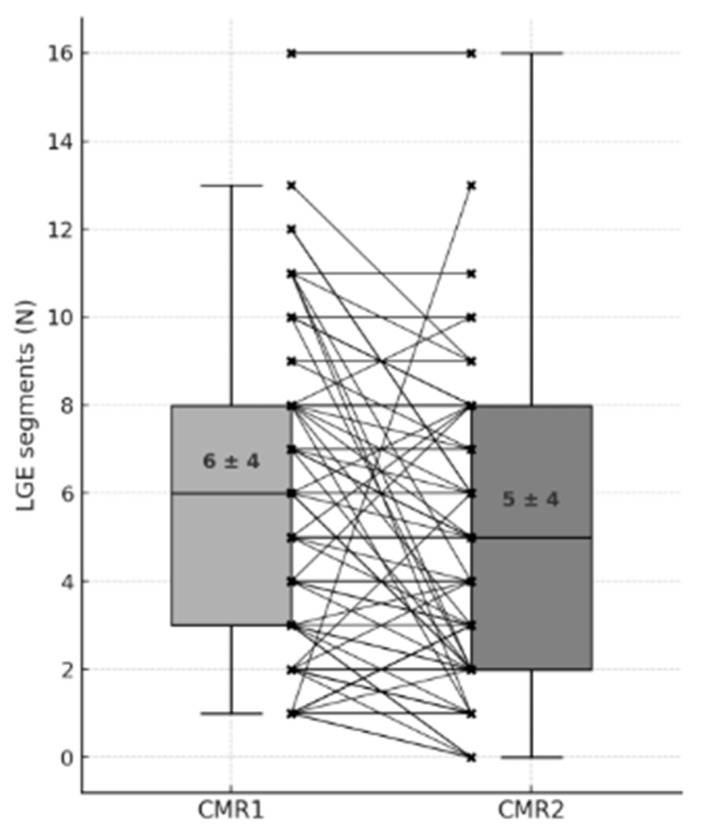
Box plot showing the number of LGE segments (N) at CMR1 and CMR2. Individual patient values are connected with black lines, illustrating paired measurements. Black dots indicate individual data points. Mean ± standard deviation (SD) is reported inside each box. The x marks indicate the extent of LGE for each patient at CMR-1 and CMR-2.

Further, we observed LGE extent reduction or disappearance in 40 patients (42%), while 49 patients showed no substantial differences in LGE compared to CMR-1 (Figure 3). Of note, an increase in LGE extent was observed in six patients (6%).

Based on CMR-2 results, 23 patients (24%) met the 2020 ESC Guidelines on CMR criteria for return-to-play after an acute myocarditis (Figure 2).

## 4. Discussion

The main findings of this study under 2020 ESC Guidelines on Sports Cardiology are as follows:

1. Most non-competitive athletes with acute myocarditis are ineligible to resume physical activity due to the presence of myocardial edema on the initial CMR scan.
2. Follow-up CMR scans at three to six months often show persistent and/or extensive LGE, with only 24% of athletes meeting the ESC Guidelines’ CMR criteria for return-to-play.
3. Septal LGE pattern is not a common finding when assessing these subjects with acute myocarditis; furthermore, none of the patients presenting with a septal LGE pattern were deemed eligible for return-to-play.

This study highlights the practical challenges physicians face when assessing athletes with myocarditis using CMR criteria. As emphasized in the 2025 ESC Guidelines for the management of myocarditis and pericarditis, current evidence on the prognostic significance of LGE extent remains limited [3]. Further research is necessary to clarify the role of LGE burden in determining outcomes for both sedentary individuals and athletes, which could lead to safer return-to-play decisions and potentially broaden eligibility for athletic participation following myocarditis.

CMR is the leading imaging modality for diagnosing acute myocarditis in hemodynamically stable patients with preserved LV systolic function, with clinical protocols recommending it within the first week of symptom onset to confirm the diagnosis using indicators like myocardial edema, LGE, and advanced mapping techniques [4]. While follow-up CMR (often at six months) monitors myocardial damage progression, the long-term implications of CMR findings remain insufficiently explored, as only a small number of studies addresses this issue. Anzini et al. demonstrated a strong correlation between LV dysfunction at admission and at six months with adverse prognosis [11], and it is well-established that the presence and extension of LGE are powerful predictors of adverse events, with midwall LGE in the interventricular septum during the acute stage suggested as a predictor of poorer long-term outcomes [12,13].

Traditionally viewed as a marker of irreversible injury [14], Mahrholdt et al. showed LGE resolution in 19 out of 71 acute myocarditis patients on repeat CMR at six months, indicating LGE may reflect acute inflammation rather than permanent fibrosis, thus highlighting the variable prognostic value of CMR indicators [15].

The ITAMY study (374 patients with acute myocarditis and preserved EF) identified the most prevalent LGE pattern as subepicardial in the LV inferior/lateral walls (IL group, 41%), while midwall LGE in the interventricular septum (AS group, 36%) was linked to worse clinical outcomes, despite similar EF, due to larger LV volumes and more extensive scarring [12].

A subsequent analysis highlighted the value of a six-month follow-up CMR, establishing persistent midwall septal LGE and LGE without accompanying edema as independent predictors of adverse events [16]. While myocardial edema resolved in 84% of patients, LGE fully disappeared in only 10%. Among those with persistent LGE, 46% showed reduction, 14% showed an increase; those with LGE but no residual edema had a worse prognosis [16]. Notably, complete LGE resolution was observed only in the subepicardial pattern, not in the midwall septal pattern [16]. The presence of increased LGE without edema at follow-up (14% of patients) was linked to poorer survival, suggesting ongoing myocardial damage, likely due to autoimmune activity or recurrent myocarditis [16].

Current evidence suggests that prognosis is determined less by the absolute extent of LGE and more by specific features, such as an anteroseptal distribution, the presence of LGE in the absence of myocardial edema, and progressive LGE expansion over time. The 2020 ESC Guidelines on Sports Cardiology recommend return-to-play in both competitive and recreational athletes after 3–6 months of physical inactivity if laboratory markers and baseline ECG normalize, absence of arrhythmias on ECG Holter monitoring and exercise testing, normal LV function and complete resolution of myocardial edema and LGE on CMR. When residual LGE persists, its extent should not exceed 20% of total myocardial mass [6]. In contrast, the 2025 ESC Guidelines for the management of myocarditis and pericarditis underscore the importance of an individualized approach, advising a minimum one-month period of exercise restriction followed by tailored reassessment [3]. Furthermore, they highlight the absence of definitive evidence regarding the long-term prognostic implications of LGE burden, thereby reinforcing the need for future studies to refine risk stratification models with a high negative predictive value for sudden cardiac death and major arrhythmic events [3].

This study highlights that, following an episode of acute myocarditis, only a minority of recreational athletes can be cleared for return-to-play based solely on cardiac magnetic resonance (CMR) criteria, without integrating other essential clinical and laboratory markers. Our understanding of the long-term prognostic significance of certain CMR features remains limited, necessitating the use of a dichotomous cut-off in current eligibility guidelines. Further elucidation of the long-term prognostic value of these findings is crucial for refining and individualizing the criteria, ultimately enabling a safe return to sport.

### Limitations

This study did not quantify the extent of LGE as a percentage of LV mass, but rather as the number of segments involved by LGE. Many different cut-off levels have been proposed in order to differentiate LGE areas from remote normal myocardium; although these approaches may produce accurate results in patients with isolated, discrete, dense scar-related LGE in ischemic cardiomyopathy, quantification is definitely less feasible in case of multiple, small, heterogeneous LGE areas such as those typically observed in myocarditis or other non-ischemic conditions [17]. In a 16-segment LV model, a >20% LV mass involvement is roughly reached with LGE extending to at least three segments. This makes CMR criteria as proposed by the COCIS recommendations comparable to those included in the 2020 ESC Guidelines and, in addition, clinically more feasible. Furthermore, this study did not consider the arrhythmic burden or other clinical parameters, as its primary objective was to offer a real-world assessment of how CMR-based ESC criteria influence the return-to-play decisions for young subjects involved in non-competitive sport activity recovering from acute myocarditis. By focusing solely on the impact of imaging criteria, we aimed to illustrate the direct consequences on athletic participation eligibility. However, expanding this research to include a larger cohort and incorporating a broader range of clinical data—such as arrhythmic events, biomarkers, exercise tolerance, genetic traits, and patient-reported outcomes—may provide a more comprehensive understanding of the long-term trajectory and risks faced by these athletes.

## 5. Conclusions

Our findings indicate that, based on CMR criteria as outlined in the latest ESC Guidelines on Sports Cardiology, only a small proportion of subjects involved in non-competitive sport activity recovering from acute myocarditis were deemed eligible to resume sport activity. This limited clearance underscores a critical gap in our understanding of the prognostic implications of CMR findings in this population. While markers such as LGE and myocardial edema provide insight into myocardial health, their exact significance in predicting long-term outcomes, functional recovery, and risks for athletes remains incompletely characterized. Future research should focus on evaluating the significance of LGE myocardial involvement over time, in terms of pattern location, extension, and evolution during follow-up. Refining and personalizing return-to-play criteria is essential to strike a balance between ensuring athlete safety, reducing the risk of adverse cardiac events, and promoting effective recovery and prompt reintegration into sport activity.

## Figures and Tables

**Table 1:** Characteristics of the study population.

95 Patients (82 m, 13 f)	CMR-1	CMR-2	*p*-Value
Age (mean)	25 (±10)	26 (±10)	
End-diastolic volume index (EDVi; mL/m^2^)	78 (±12)	78 (±11)	0.9
Ejection fraction (EF)	65% (±6)	66% (±6)	0.67
EF < 55% (N. pts)	2	3	0.65
LGE extent (N. LV segments)	6 (±4)	5 (±4)	0.1
LGE septal involvement (N. pts)	10 (11%)	11 (11%)	0.81
Edema (STIR+) (N. pts)	80 (84%)	12 (13%)	<0.001
Reduced LGE (N. pts)	/	40 (42%)	
Unchanged LGE (N. pts)	/	49 (51%)	
Increased LGE (N. pts)	/	6 (6%)	
Eligible by ESC 2020 Guidelines (N. pts)	/	23 (24%)	

LGE = late gadolinium enhancement; STIR = short tau inversion recovery.

## Data Availability

The data presented in this study are available upon reasonable request from the corresponding author. The data are not publicly available due to privacy concerns.

## Abbreviations

The following abbreviations are used in this manuscript:

CMR	Cardiovascular magnetic resonance
b-SSFP	Balanced steady-state free-procession
LGE	Late gadolinium enhancement
LV	Left ventricular/left ventricle
EF	Ejection fraction
STIR	Short tau inversion recovery
ESC	European Society of Cardiology
EDVi	End-diastolic volume index
ICD	Implantable cardioverter–defibrillator

## References

[1] Aretz H.T., Billingham M.E., Edwards W.D., Factor S.M., Fallon J.T., Fenoglio J.J., Olsen E.G., Schoen F.J. (1987). Myocarditis. A histopathologic definition and classification. Am. J. Cardiovasc. Pathol..

[2] Ammirati E., Raimondi F., Piriou N., Sardo Infirri L., Mohiddin S.A., Mazzanti A., Shenoy C., Cavallari U.A., Imazio M., Aquaro G.D. (2022). Acute Myocarditis Associated with Desmosomal Gene Variants. JACC Heart Fail..

[3] Schulz-Menger J., Collini V., Gröschel J., Adler Y., Brucato A., Christian V., Ferreira V.M., Gandjbakhch E., Heidecker B., Kerneis M. (2025). 2025 ESC Guidelines for the management of myocarditis and pericarditis. Eur. Heart J..

[4] Ferreira V.M., Schulz-Menger J., Holmvang G., Kramer C.M., Carbone I., Sechtem U., Kindermann I., Gutberlet M., Cooper L.T., Liu P. (2018). Cardiovascular Magnetic Resonance in Nonischemic Myocardial Inflammation: Expert Recommendations. J. Am. Coll. Cardiol..

[5] Halle M., Binzenhöfer L., Mahrholdt H., Johannes Schindler M., Esefeld K., Tschöpe C. (2021). Myocarditis in athletes: A clinical perspective. Eur. J. Prev. Cardiol..

[6] Pelliccia A., Sharma S., Gati S., Bäck M., Börjesson M., Caselli S., Collet J.P., Corrado D., Drezner J.A., Halle M. (2021). 2020 ESC Guidelines on sports cardiology exercise in patients with cardiovascular disease. Eur. Heart J..

[7] Zeppilli P., Biffi A., Cammarano M., Castelletti S., Cavarretta E., Cecchi F., Colivicchi F., Contursi M., Corrado D., D’Andrea A. (2024). Italian Cardiological Guidelines (COCIS) for Competitive Sport Eligibility in athletes with heart disease: Update 2024. Minerva Med..

[8] Rubino M., Scatteia A., Frisso G., Pacileo G., Caiazza M., Pascale C.E., Guarini P., Limongelli G., Dellegrottaglie S. (2021). Imaging the “Hot Phase” of a Familiar Left-Dominant Arrhythmogenic Cardiomyopathy. Genes.

[9] Kramer C.M., Barkhausen J., Bucciarelli-Ducci C., Flamm S.D., Kim R.J., Nagel E. (2020). Standardized cardiovascular magnetic resonance imaging (CMR) protocols: 2020 update. J. Cardiovasc. Magn. Reson..

[10] Schulz-Menger J., Bluemke D.A., Bremerich J., Flamm S.D., Fogel M.A., Friedrich M.G., Kim R.J., von Knobelsdorff-Brenkenhoff F., Kramer C.M., Pennell D.J. (2020). Standardized image interpretation and post-processing in cardiovascular magnetic resonance—2020 update: Society for Cardiovascular Magnetic Resonance (SCMR): Board of Trustees Task Force on Standardized Post-Processing. J. Cardiovasc. Magn. Reson..

[11] Anzini M., Merlo M., Sabbadini G., Barbati G., Finocchiaro G., Pinamonti B., Salvi A., Perkan A., Di Lenarda A., Bussani R. (2013). Long-term evolution and prognostic stratification of biopsy-proven active myocarditis. Circulation.

[12] Aquaro G.D., Perfetti M., Camastra G., Monti L., Dellegrottaglie S., Moro C., Pepe A., Todiere G., Lanzillo C., Scatteia A. (2017). Cardiac MR with Late Gadolinium Enhancement in Acute Myocarditis with Preserved Systolic Function: ITAMY Study. J. Am. Coll. Cardiol..

[13] Eichhorn C., Greulich S., Bucciarelli-Ducci C., Sznitman R., Kwong R.Y., Gräni C. (2022). Multiparametric Cardiovascular Magnetic Resonance Approach in Diagnosing, Monitoring, and Prognostication of Myocarditis. J. Am. Coll. Cardiol. Imaging.

[14] Friedrich M.G., Sechtem U., Schulz-Menger J., Holmvang G., Alakija P., Cooper L.T., White J.A., Abdel-Aty H., Gutberlet M., Prasad S. (2009). Cardiovascular magnetic resonance in myocarditis: A JACC White Paper. J. Am. Coll. Cardiol..

[15] Mahrholdt H., Wagner A., Deluigi C.C., Kispert E., Hager S., Meinhardt G., Vogelsberg H., Fritz P., Dippon J., Bock C.T. (2006). Presentation, patterns of myocardial damage, and clinical course of viral myocarditis. Circulation.

[16] Aquaro G.D., Ghebru Habtemicael Y., Camastra G., Monti L., Dellegrottaglie S., Moro C., Lanzillo C., Scatteia A., Di Roma M., Pontone G. (2019). Prognostic Value of Repeating Cardiac Magnetic Resonance in Patients with Acute Myocarditis. J. Am. Coll. Cardiol..

[17] Gräni C., Eichhorn C., Bière L., Kaneko K., Murthy V.L., Agarwal V., Aghayev A., Steigner M., Blankstein R., Jerosch-Herold M. (2019). Comparison of myocardial fibrosis quantification methods by cardiovascular magnetic resonance imaging for risk stratification of patients with suspected myocarditis. J. Cardiovasc. Magn. Reson..

